# How Much of the Success of Filling Teeth Is Due to the Manipulation

**Published:** 1874-01

**Authors:** H. A. Smith


### HOW MUCH OF THE SUCCESS OF FILLING TEETH IS DUE TO THE MANIPULATION?

*Subject for Discussion before the Ohio State Dental Society.*

BY H. A. SMITH.

Dr. Robert Arthur, in the preface of his work upon the "*Treatment and Prevention of decay of the Teeth,*" says: "The great object of dental surgery is, of course, the preservation of the natural teeth. Yet it cannot be denied by those who have given most attention to the matter that it falls, in this respect, when the general result is considered, far short of what it might reasonably be expected to accomplish." In confirmation of this assertion, he states that the number of teeth extracted annually in the United States is estimated at twenty millions. He further says that "in a great many cases in which the teeth have been finally destroyed by decay, attempts have been made to save them" by filling the decayed cavities with gold or other materials. To show the extent of the practice of filling teeth in our own country, he gives the estimate of the value of gold foil used annually for this purpose at three million dollars. The wholesale loss of teeth to which he calls attention would indicate, he remarks, "It is certain either the means relied upon for the arrest of decay are insufficient for the purpose, or they are imperfectly applied."

Dr. Arthur has had large opportunity for observation, and his statements should command the earnest attention of the dental practitioner. But, when he states that the object of his book "is to describe a new method of treating teeth," by which "*all the teeth of every individual, with few exceptions, may be preserved,*" it may be proper to suggest that his zeal in advocating his "new" and "more efficient means" of relief from dental caries may have influenced him to depreciate the real value of the usual operation of filling carious teeth. This author's strictures, however, upon the every-day practice furnish food for serious reflection. And the subject now under discussion; viz., How much of the success in filling teeth is due to the manipulation? is one that may be profitably considered.

What then is the true measure of success in filling teeth? Is it that the tooth affected shall be preserved during the life of the individual, and under all circumstances? Whilst at first view this would not seem an unreasonable test, especially if applied by the patient himself, yet the dentist would hardly be willing to have his reputation as a successful practitioner tried bv this standard. Every intelligen dentist knows that there is a number of modifying circumstances, such as the laws of heredity, sex, age, temperament, occupation, habits of cleanliness, etc., which will to a less or greater extent influence the ultimate success of nearly every case of filling teeth. And the nature of the operation itself, when we consider that it is performed upon a vital tissue, and often for highly nervous and shrinking persons, would preclude the idea of permanently saving the teeth in all cases. The evidence which we have of a predisposition to disease or that there is some local or general agency at work to induce caries in a tooth, in the fact that it requires this surgical and mechanical treatment at our hands, would still further indicate that the success of the operation does not alone depend upon the mere manipulation. Let a tooth never so well filled be subject to precisely the same conditions that induced disorganization of the bone in the first instance, and the disease may reappear in near proximity to the filling. Again: it is quite probable that caries in a tooth may not be arrested perceptibly at all, unless a more favorable state of the general health is brought about, or a change in the secretions of the mouth from a morbid to a healthy condition is effected either before or soon after the operation of filling.

But whilst it is true, as I have stated, that many of the cases of failure in filling teeth may be traced directly to causes that are not fully under the control of the dentist, yet, in the larger number of cases, where dental caries has not been permanently arrested by filling, the failure has been caused by faulty manipulation, either in the preparation of the cavity or in the introduction of the material for the filling. Or it may be, if the operation is made in the proximate surface of the tooth, that the proper relation of the surfaces has not been observed, so that the necessary cleansing of the parts could be most readily effected.

These are truths, humiliating though they be, that are brought to our attention almost daily, by observing our own failures as well as those of others, who are accounted most skilled in manipulation.

Notwithstanding the boasted improvements in operating of late years,—and they have been marked and radical,— the majority of operators fall far short, even when a legitimate test is applied, in the number of cases in which they have succeeded in permanently arresting caries of the teeth by this method of treatment. Not a few of these failures may be attributed to gross ignorance of the correct principles to be observed in filling teeth, and the want of proper training in the manipulation on the part of the dentist who has had the case in charge. Others, more capable, excuse themselves for not putting forth their best efforts, on the ground that circumstances are not favorable, that the patient does not appreciate the best class of operations, or that he is unwilling to pay for them. And it is owing to these and similar practices that the general profession is subjected to the frequent charge, that they claim too much for filling as a means of saving the teeth.

It must be apparent to all, that the largest degree of success in operative dentistry can only be obtained by combining with skill and tact in manipulation, a correct knowledge—at least so far as investigation has given us light upon the subjects—of the etiology and pathology of dental caries. The dentist who unites these attainments in the greatest measure invariably exhibits a zeal and enthusiasm in the direction of conservative dentistry, which is in turn imparted, in some degree, to the patient ; and without which the dentist would not succeed nearly so well in difficult cases, nor the patient give that care and attention to his teeth which is so essential to the permanency of this class of operations, no matter how perfect in character.

We may well take pride in what our profession has done for mankind in the direction of saving the natural teeth. But dental science will fall far short of its true mission until it shall be able to discover to us with greater certainty the more active causes which induce dental caries—whereby we may be able to prescribe a course of hygienic or prophylactic treatment which shall render the disagreeable and painful operation of filling teeth much less frequent than the demands of the present generation require.

